# Response of cells and tissues to shear stress

**DOI:** 10.1242/jcs.260985

**Published:** 2023-09-25

**Authors:** Jaime A. Espina, Marilia H. Cordeiro, Milan Milivojevic, Ivana Pajić-Lijaković, Elias H. Barriga

**Affiliations:** ^1^ Mechanisms of Morphogenesis Lab, Gulbenkian Institute of Science (IGC), 2780-156 Oeiras, Portugal; ^2^ Faculty of Technology and Metallurgy, Belgrade University, 11120 Belgrade, Serbia

**Keywords:** Shear stress, Fluid shear stress, Biomechanics, Cytoskeleton

## Abstract

Shear stress is essential for normal physiology and malignancy. Common physiological processes – such as blood flow, particle flow in the gut, or contact between migratory cell clusters and their substrate – produce shear stress that can have an impact on the behavior of different tissues. In addition, shear stress has roles in processes of biomedical interest, such as wound healing, cancer and fibrosis induced by soft implants. Thus, understanding how cells react and adapt to shear stress is important. In this Review, we discuss *in vivo* and *in vitro* data obtained from vascular and epithelial models; highlight the insights these have afforded regarding the general mechanisms through which cells sense, transduce and respond to shear stress at the cellular levels; and outline how the changes cells experience in response to shear stress impact tissue organization. Finally, we discuss the role of shear stress in collective cell migration, which is only starting to be appreciated. We review our current understanding of the effects of shear stress in the context of embryo development, cancer and fibrosis, and invite the scientific community to further investigate the role of shear stress in these scenarios.

## Teaser

**Summary:** We discuss shear stress in response to fluid dynamics and invite the reader to study shear stress in biological systems, in which shear stress emerges from cell and tissue interactions.

## Introduction

Shear stress corresponds to the deformation of an object resulting from the application of a force tangential to its surface. Biological systems appear to be more sensitive to shear stress than to other types of stress. For instance, the mechanical and transcriptional state of epithelial cells can be influenced by shear stresses of just a few pascals (Flitney et al., 2009), whereas the same cells are unaffected by compressive stress (see Glossary) in the order of kilopascals (Tse et al., 2012; Cheng et al., 2009). Shear stress is generated spontaneously in biological systems, such as in vascular endothelial cells (Box 1) due to the frictional force exerted by the blood flow, in glomerular endothelial cells due to the flow induced by glomerular filtrate, in intestinal epithelial cells due to peristaltic movements, in corneal cells due to eye blinking, and in collectively migrating cells (such as metastatic cancer cells) or cells moving in tissues during development due to friction between cell layers. In any of these cellular models, cells can sense and respond to shear stress. The response to shear stress can vary on a temporal scale, ranging from rapid protein modifications that occur in the order of milliseconds to seconds and that typically modulate cell behavior, to long-term transcriptional changes that can even modify cell fate (Baeyens et al., 2016; Macek Jilkova et al., 2014; Stolberg and McCloskey, 2009). Here, we start by addressing the role of fluid shear stress (FSS; see Glossary) in endothelial and epithelial cells, two of the most studied models of shear stress, before outlining how FSS can be sensed and transduced to elicit a cellular response. Next, we focus on the roles of non-fluid FSS in collective cell migration, addressing how it arises in this context, what the possible roles of non-fluid FSS are and how it can be estimated using mathematical modeling. Finally, we highlight pathologies that might be caused by non-physiological levels of shear stress.

**Glossary**

**Atheroprotection:** protection against atherosclerosis.

**Azimuthal angle and radial distance:** coordinates of the polar coordinate system, which is suitable for describing two-dimensional circular movement.

**Azimuthal shear rate:** radial gradient of the azimuthal velocity generated during cell swirling motion.

**Azimuthal velocity:** depends on the radial distance and the change of the azimuthal angle with time.

**Compressive stress:** a type of normal stress caused by shortening of one, two or three dimensions. This stress can induce a decrease in the object volume, depending on its rheological behavior.

**Contact inhibition of locomotion:** the process where two migratory cells stop their migration upon collision with each other and repolarize in order to migrate in opposite directions.

**EMT:** epithelial-to-mesenchymal transition; the process by which cells can transit from an epithelial-like phenotype to a mesenchymal-like phenotype.

**Fluid shear stress:** a type of stress that induces a shear flow of fluid.

**Mechanical stress:** physical quantity that describes the magnitude of forces per unit area that cause a deformation.

**Normal stress:** stress that acts perpendicular to the surface of an object. It can be extensional or compressional.

**Residual shear stress:** the stress that remains in any material even in absence of external forces and represents the sum of normal and shear stresses.

**Shear rate:** the rate of change in velocity at which one surface passes over an adjacent surface.

**Shear stress:** a type of stress that acts coplanar with the cross section of an object. This stress is orthogonal to normal stress.

**Slip effects:** an event characterized by a part of fluid in motion not adhering to a substrate. This event generates a difference in velocity between the non-adherent area and the rest of the material.

**Stress relaxation:** observed, time-dependent decrease in the stress of an object under constant strain.

**Torque:** a moment of shear stress that twists the structure or induces swirling motion of soft matter systems.

**Box 1. Shear stress in physiological contexts**

In humans, normal physiological blood flow generates a range of shear stress of 0.1–9.5 Pa (Baeyens et al., 2016; Koutsiaris et al., 2007; Roux et al., 2020). However, the obtained value for any structure is only an average, dependent on the position where the flow has been measured and the mode of calculation. Hence, any value from the literature and given here should be taken as a general order of magnitude rather than an actual absolute value for a given structure. In addition, different types of blood vessels present distinct shear stress signatures induced by blood flow. For instance, as blood movement is caused by beating of the heart, the FSS exhibited in arteries is highly oscillatory, pulsatory in synchrony with the heartbeat and, with average values of 1–2 Pa, likely dependent on the size of the vessel. Moving through the circulatory system away from the heart, in arterioles, oscillations in shear stress decrease, but the magnitude of the stress is greater, with an average of 5–8 Pa. In the smaller arterioles and capillaries, shear stress even reaches values up to 9.5 Pa (Koutsiaris et al., 2007) with only minimal oscillations. Then, as the vessel starts growing again in the venous system, the registered mean values are between 2 and 4 Pa in small venules and up to 0.3 Pa in postcapillary venules (Ballermann et al., 1998; Koutsiaris et al., 2007). Finally, in veins, the average shear stress values range from 0.1 to 0.6 Pa (Baeyens et al., 2016; Paszkowiak and Dardik, 2003).

Another physiological context where shear stress is generated is the intestine. Here, peristaltic movements due to organ contractility allow particles to flow, generating oscillatory shear stress in the epithelial cells of the intestine surface. The shear stress values estimated in these surfaces range from 0 to 0.003 Pa (Delon et al., 2019; Lentle and Janssen, 2008). Finally, epithelial cells in the cornea experience shear stress due to blinking of the eye; here, shear stress is pulsatory and has been proposed to be in the range of 0.005–1.5 Pa (Kang et al., 2014; Srinivas et al., 2002).

## Fluid shear stress in vascular endothelial cells

**Fig. 1.**
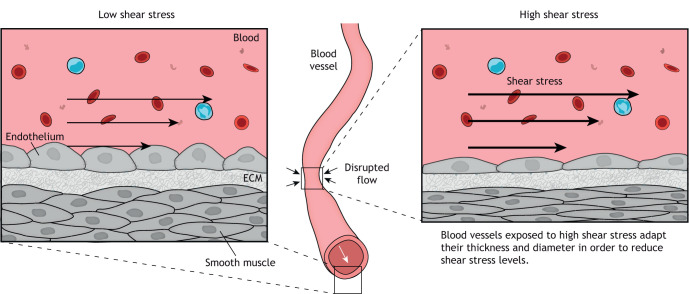
**Schematic illustration of how shear stress can affect blood vessels.** Blood flow generates shear stress at the surface of the endothelial cells lining the blood vessel, to which the vessel responds to maintain normal shear stress levels. Under low physiological levels of shear stress, the vessel increases its thickness (left), specifically of the tunica media, and so decreases the internal diameter. This is achieved by thickening of the cellular body and loss of cell polarity. In areas of higher stress, such as in constrictions, the thickness of the vessel is reduced, and thus the internal diameter is increased (right). This occurs via a cellular mechanism involving elongation of cells and polarization of the cells in the direction of blood flow through activation of pathways that promote vasodilation.

The most common physiological FSS arises at the interface between blood and blood vessels, which are lined with a single layer of endothelial cells. The magnitude of this type of shear stress depends on blood velocity and viscosity, as well as on the diameter of the vessel. Blood flow through vessel regions with a reduced diameter (arterioles, capillaries and venules) results in a higher average blood velocity and higher shear rate (see Glossary) near the blood vessel wall, compared to that in wider vessels (arteries and veins) (Fig. 1; Box 1). Physiological shear stress caused by blood flow represents an optimal environment for the endothelial monolayer (Baeyens et al., 2016) (Box 1), promoting cell elongation and an alignment of cell polarity with the direction of flow; this in turn suppresses proliferation and stimulates the expression of anti-inflammatory genes, preventing activation of inflammatory pathways (Roux et al., 2020). An extensive body of work has addressed the role of FSS in different aspects of endothelial cell biology (Souilhol et al., 2020; Roux et al., 2020; Chistiakov et al., 2017). Here, we highlight only some of the general aspects of the effect of FSS on blood vessels for further discussion.

In response to physiological variations in shear stress, such as variations of heart rate due to exercise, cells are capable of self-regulating the FSS to some extent (Roux et al., 2020) (Fig. 1). At the cellular level, a local decrease in FSS promotes changes in cell shape, such as a decrease in cell polarity and thickening of the cellular body (Inglebert et al., 2020; Zhou et al., 2012). These changes may be sufficient to reduce the internal diameter of the blood vessel, which would then increase the FSS to the optimum value (Langille et al., 1989; Chistiakov et al., 2017). In contrast, a local increase in FSS results in cell polarization against the lumen of the vessel, with a resulting elongation of cells and nuclear shrinkage (Baeyens et al., 2016; Jetta et al., 2019; Zhou et al., 2014). In addition, FSS can activate pathways that promote nitric oxide (NO) production, a known vasodilator and a signature molecule induced by shear stress in endothelial cells (Pahakis et al., 2007); in turn, these adaptations can result in a lowering of the FSS that cells experience (Chistiakov et al., 2017). However, endothelial cells are not only sensitive to variations in the magnitude of shear stress; they can also differentially respond to a change in the direction of the shear stress vector (Givens and Tzima, 2016). For instance, directional alteration of FSS can result in changes to cell morphology and blood vessel permeability, as well as weakening or reducing cell–cell adhesion contacts and inducing inflammatory responses (Chistiakov et al., 2017; Givens and Tzima, 2016).

FSS has also been shown to be required for remodeling and morphogenesis of several tissues within the vascular system during development and disease (Baeyens et al., 2016; Boselli et al., 2017; Chen and Tzima, 2009; Groenendijk et al., 2004; Langille et al., 1989; Lucitti et al., 2007; Tzima et al., 2005; Zhou et al., 2014). For instance, shear stress can be reduced by decreasing the percentage of blood volume made up of blood cells, which in turn decreases expression of nitric oxide synthase 3 (*NOS3*), a gene related to NO production and vessel remodeling. Indeed, the dependence of vascular remodeling on shear stress has been extensively demonstrated (Baeyens et al., 2016; Chen and Tzima, 2009; Langille et al., 1989; Tzima et al., 2005; Zhou et al., 2014). In addition, FSS arising from myocardial contractility and blood flow is essential for heart morphogenesis (Boselli et al., 2017; Groenendijk et al., 2004; Lucitti et al., 2007). Indeed, many genes known to be regulated by FSS – such as krueppel-like factor 2 (*KLF2*), which is known to contribute to endothelium maintenance and vasorelaxation, and have anti-inflammatory effects; endothelin-1 (*ET-1*), a known vasoconstrictor and pro-inflammatory factor; and *NOS3* – are expressed during heart development in regions of high shear stress (Groenendijk et al., 2004). Moreover, during morphogenesis of the heart valve, endothelial cells can modulate their migration trajectories in response to blood flow-triggered shear stress (Boselli et al., 2017).

## Fluid shear stress in epithelial cells

Similar to endothelial cells in blood vessels, epithelial cells in tissues, such as the intestine or cornea, can be exposed to FSS. The effect of FSS on monolayers of the epithelial-like intestinal cell line Caco-2 has been investigated (Delon et al., 2019). Exposure of a 10 µm-thick Caco-2 cell monolayer to a range of physiological levels of FSS has been reported to result in an increase in thickness of about threefold in cells exposed to the highest FSS levels (Delon et al., 2019) (Box 1). Similarly, corneal cells exposed to different levels of physiological FSS show modifications at both the subcellular and cellular levels (Molladavoodi et al., 2017). Exposure to different magnitudes of FSS have also been used to test wound healing (Molladavoodi et al., 2017; Albuquerque et al., 2000). For instance, Molladavoodi et al. (2017) have reported that cells primed with low levels of FSS show improved wound closure compared to cells that experience static FSS or that are only exposed to FSS after a scratching assay. This improved wound healing has been shown to be due to an increase in rates of cell migration and proliferation within 24 h of exposure, whereas FSS greater than physiological values induces cell damage and extensive apoptosis (Molladavoodi et al., 2017). However, these assays do not reflect the *in vivo* context, and therefore, tools have been developed to more closely recreate physiological conditions (Lin et al., 2019). Finally, *in vivo* experiments using wound healing assays in rabbit ear chambers have demonstrated that ear microvessels heal faster when the tissue is exposed to shear stress (Ichioka et al., 1997).

One major adaptation of epithelial cells to shear stress is the induction of epithelial-to-mesenchymal transition (EMT; see Glossary), whereby epithelial cells acquire a mesenchymal phenotype and behavior after the downregulation of epithelial features (Yang et al., 2020). Indeed, it has been observed that FSS can induce EMT in adenocarcinoma cell lines (Liu et al., 2016) and in ovarian cancer (Rizvi et al., 2013), but the underlying molecular mechanisms have been elusive.

## Sensing, transduction and subcellular responses to fluid shear stress

Maintaining tissue homeostasis relies on the ability of cells to respond and adapt to a variety of biochemical and mechanical signals from the environment, and the failure to adapt to such challenges, in this case variations in shear stress, can lead to tissue damage. In order to elicit a change in a cell, shear stress must first be detected at the cell surface, through mechanosensing, and then be translated into a biochemical signal, in a process referred to as mechanotransduction. There are several excellent summaries of the general aspects of mechanosensing and mechanotransduction (Martino et al., 2018; Salvador and Iruela-Arispe, 2022; Yap et al., 2018); hence, we will specifically focus on the subcellular components involved in sensing and transduction of FSS.

### Membrane

**Fig. 2.**
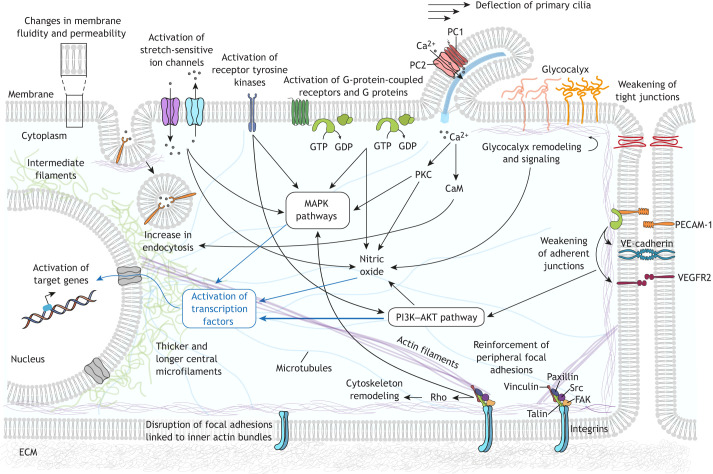
**Cellular responses to shear stress.** Simplified representation of the molecular mechanisms involved in mechanosensing and mechanotransduction triggered in different cell compartments in response to shear stress. At the membrane, shear stress induces changes in fluidity and permeability, which can lead to activation of membrane-bound molecules, including activation of G proteins independently of the G-protein-coupled receptors. Membrane-bound receptors (such as receptor tyrosine kinases and G-protein-coupled receptors) and stretch-activated ion channels (such as PIEZO1) can also be activated by shear stress. FSS can bend primary cilia and trigger Ca^2+^ influx through activation of polycystin-1 (PC1) and polycystin-2 (PC2) transmembrane proteins. This rise in intracellular levels of Ca^2+^ can activate multiple signaling molecules and induce an increase in endocytosis of proteins and small molecules. Shear stress causes conformational changes in the glycocalyx, affecting the local concentration of molecules in the extracellular domain and inducing several signaling pathways in the intracellular domain, including those regulating the production of NO and the organization of the cytoskeleton. Activation of tyrosine kinases in FAs, such as FAK and Src, leads to activation of multiple signaling molecules and cytoskeleton reassembly in response to shear stress. FSS leads to a remodeling of FAs that is dependent on the stability of the actin stress fibers linked to them: disassembly of FAs anchored to inner perpendicular fibers is promoted, whereas FAs linked to peripheral fibers remain stable. Shear stress is associated with weakening of both tight junctions and adherent junctions. Adhesion proteins (such as PECAM-1 and VE-cadherin) in cell–cell junctions can sense and respond to shear stress, causing activation of VEGFR2 tyrosine kinase and induction of downstream signaling cascades such as the PI3K–AKT pathway. In response to shear stress, cytoskeletal fibers suffer major modifications and rearrangements. In the cytoplasm, mechanosensors induce activation of multiple signaling molecules and cascades, including PI3K–AKT and mitogen-activated protein kinase (MAPK) pathways. This causes several transcription factors (such as NRF2 and NF-κB) to be translocated to the nucleus, where they alter gene expression, leading to long-term cellular adaptations necessary to cope with different levels of shear stress. CaM, calmodulin; PKC, protein kinase C; Rho, Ras homolog proteins.

The first cellular component experiencing external stress is the cell membrane (White and Frangos, 2007). Here, the glycocalyx, the membrane itself and membrane-bound factors have been shown to be the primary hub for shear stress sensing (Fig. 2).

The glycocalyx is a dense macromolecular mesh composed of proteoglycans and glycosaminoglycans decorating the endothelial outer membrane, and has been shown to be both a primary mechanosensor and final effector for cellular adaptation to FSS (Pahakis et al., 2007; Psefteli et al., 2021; Wang et al., 2020; Zeng and Tarbell, 2014; Zeng et al., 2018; Barvitenko et al., 2022) (Fig. 2). For instance, in bovine aortic endothelial cells exposed to FSS, depletion of a specific glycocalyx component has been shown to affect NO production, lowering NO levels to those observed in cells exposed to static conditions (Pahakis et al., 2007). In the same manner, in an *ex vivo* approach, experiments performed in isolated vessels have shown that NO production is affected after glycocalyx alterations (Hecker et al., 1993; Mochizuki et al., 2003). More recently, glycocalyx disruption in human endothelial cells has been found to attenuate the activation of nuclear factor erythroid 2-related factor 2 (NRF2), a transcription factor important in the response to oxidative stress caused by reactive oxygen species (ROS) that is activated by laminar shear stress (Psefteli et al., 2021; Zakkar et al., 2009), linking FSS to ROS production. Glycocalyx components have also been shown to act as final effectors in adaptation to FSS (Psefteli et al., 2021; Zeng and Tarbell, 2014). For instance, oscillatory shear stress, but not laminar flow shear stress, impairs the glycocalyx in human endothelial cells (Psefteli et al., 2021).

FSS is able to increase both the permeability and fluidity of the membrane (Berthiaume and Frangos, 1994; Haidekker et al., 2000) (Fig. 2). Interestingly, increases in membrane fluidity have been shown to activate G proteins in liposomes in the absence of G-protein-coupled receptors (Gudi et al., 1998). Thus, it has been speculated that the first step of the mechanosensitive response to shear stress arises from changes in membrane fluidity, leading to activation of G proteins (White and Frangos, 2007). However, other studies have reported the activation of G proteins as a mediator of shear stress-induced responses (Berthiaume and Frangos, 1992; Kuchan et al., 1994) downstream of other mechanosensors (discussed further below).

Sensitive ion channels, such as the stretch-activated cation channel PIEZO1, are also involved in shear stress mechanosensing and cellular adaptation (Dela Paz and Frangos, 2019; Iring et al., 2019). For instance, laminar shear stress can activate PIEZO1, inducing the release of adrenomedullin, which is a known hormone vasodilator, from the exposed cells. Adrenomedullin can act then in an autocrine and paracrine manner to activate G proteins inside the cell and so modulates mechanotransduction pathways, ultimately leading to vasodilation mediated by NOS production (Iring et al., 2019). However, the role of PIEZO1 as a primary sensor of shear stress is still controversial, as other work has suggested that PIEZO1 is activated downstream of G protein activation owing to shear stress rather than upstream (Dela Paz and Frangos, 2019).

Primary cilia that protrude from endothelial cells can also act as mechanosensors (Fig. 2). Primary cilia are restricted to regions of low and oscillatory shear stress *in vivo* and have been shown to confer sensitivity to shear stress in endothelial cells (Hierck et al., 2008; Van der Heiden et al., 2006). Mechanosensing by primary cilia requires polycystin-1, an atypical G-protein-coupled receptor, and polycystin-2, a non-selective Ca^2+^-permeable channel, which has been shown to mediate an increase in intracellular Ca^2+^, NO release and protein modifications in response to FSS, both in normal endothelial cells and in vascular disorders (AbouAlaiwi et al., 2009; Nauli et al., 2008; Pala et al., 2018).

Another process involved in cellular adaptation to shear stress is endocytosis. Several cell types have been shown to modify their endocytic ability in response to shear stress, in both physiological and pathological contexts (He et al., 2018; Lawler et al., 2009; Long et al., 2017; Lopez-Hernandez et al., 2020) (Fig. 2). For instance, in the kidney, there is a mechanical interaction between glomerular cells, which are responsible for filtering, and the cells at the surface of the proximal tubule, which recover some of the proteins and small molecules from the filtrate (Long et al., 2017). In this system, the endocytic capacity of the tubular cells is dependent on the level of shear stress that is induced by the flow of the glomerular filtrate (Long et al., 2017). Here, it has been proposed that shear stress can induce bending of cilia, which might, in turn, promote an increase in intracellular levels of Ca^2+^ by increasing influx owing to release from the endoplasmic reticulum (Long et al., 2017). Such an Ca^2+^ increment can induce the activation of Cdc42 through calmodulin, which might, in turn, facilitate endocytosis by promoting actin polymerization (Bhattacharyya et al., 2017; Boulant et al., 2011). This change in endocytic capacity correlates with the abovementioned changes in cell surface characteristics such as membrane fluidity.

### Cell–cell adhesions

One of the best studied mechanosensors of FSS is the endothelial junctional complex formed by PECAM-1, vascular endothelial growth factor receptors (specifically VEGFR2, also known as KDR) and vascular endothelial cell cadherin (VE-cadherin, also known as CDH5) (Fig. 2). Upon shear stress exposure, endothelial cells increase tension across PECAM-1. PECAM-1 is a cell–cell adhesion and signaling molecule known to have diverse roles in vascular biology, and it can bind to cytoskeletal components, such as vimentin intermediate filaments (VIFs) (Woodfin et al., 2007). Shear stress increases the association of PECAM-1 with vimentin, allowing the tension exerted on PECAM-1 to be transmitted along these VIFs and across the cell (Conway et al., 2013; Conway and Schwartz, 2015; Osawa et al., 2002). This can activate the Src family of tyrosine kinases, with subsequent phosphorylation of VEGFR2, leading to activation of the phosphoinositide 3-kinase (PI3K)–AKT pathway (Tzima et al., 2005). In contrast, VE-cadherin in static conditions is under high levels of myosin-dependent tension. When shear stress is applied, actomyosin filaments seem to relax, leading to a decrease in the tension applied to VE-cadherin, which then promotes VEGFR2 activation by acting as an adapter that interacts with the cytoplasmic domain of VEGFR2 (Coon et al., 2015; Tzima et al., 2005). This has also been investigated *in vivo* by analyzing vessels from PECAM-1-deficient mice, which exhibit an impairment in vascular remodeling in response to FSS that is mediated by the AKT pathway (Chen and Tzima, 2009). PECAM-1 has also been demonstrated to have a negative role in arteriogenesis and remodeling of collaterals (new arteries that grow to restore blood flow after an occlusion) (Chen et al., 2010). Along the same lines, other groups have shown that knockout of PECAM-1 is linked to a variety of phenotypes that are associated with progression of atherosclerosis (Goel et al., 2008; Lertkiatmongkol et al., 2016; Stevens et al., 2008).

PECAM-1 can also form a complex with the Gα_q_ (GNAQ) and Gα_11_ (GNA11) subunits (here referred to collectively as Gα_q/11_) of heterodimeric G proteins (Fig. 2), which dissociates when cells are exposed to oscillatory FSS, with subsequent relocalization of the G subunit to perinuclear regions (Otte et al., 2009; Yeh et al., 2008). Interestingly, knockdown of single Gα proteins in endothelial cells has little effect, but the combined depletion of Gα_i_ (GNAI) and Gα_q/11_ inhibits all known PECAM-1-dependent responses (Tanaka et al., 2020, preprint). Thus, the PECAM-1–Gα_q/11_ complex has been proposed to be another mechanosensor of shear stress; however, the exact role of this complex and possible interactions with other G proteins and PECAM complexes remain to be explored.

Cell–cell junction remodeling is another mechanism of cellular response to shear stress. For instance, when porcine carotid arterial endothelial cells are exposed to low levels of FSS (Box 1), an increase in blood vessel permeability can be observed due to weakening of tight junctions after 12 h of exposure to FSS (Conklin et al., 2007) (Fig. 2). In accordance, a later study testing the effect of FSS on cell–cell adhesion contacts within a collectively migrating endothelial monolayer has reported an FSS-induced weakening of adherens junctions that results in a reduction of the normal stress (Box 1) between cells (Steward et al., 2015) (Fig. 2). Interestingly, a comparison between the effects of low oscillatory FSS versus static FSS of the same value has found that oscillatory stress can induce an increase in the expression of E-cadherin (CDH1) and the tight junction protein occludin (Berardi and Tarbell, 2009), suggesting a reinforcement of adherent and tight junctions under oscillatory stress.

### Focal adhesions

At their surface, cells are anchored to the extracellular matrix (ECM) through focal adhesions (FAs), large integrin-containing protein complexes that link the intracellular actin cytoskeleton to the ECM (Mavrakis and Juanes, 2023). FA proteins have been shown to be important for mechanosensing and transduction of shear stress (Braddock et al., 1998; Shyy and Chien, 2002) (Fig. 2). For instance, shear stress can promote activation of β1-integrins and, in turn, activation of focal adhesion kinase (FAK, also known as PTK2) (Macek Jilkova et al., 2014; Li et al., 1997). FA dynamics depend on both the mechanical stress (see Glossary) experienced by the cell and the stability of the actin cytoskeleton (which can also be affected by external mechanical stresses such as shear stress; see below). Along these lines, it has been shown that exposing Madin–Darby canine kidney (MDCK) type II cells to low levels of FSS induces changes in FA distribution that are dependent on actin stability (Verma et al., 2015) (see below). Taken together, and like other subcellular elements, FAs are part of the mechanosensing, mechanotransduction and effector machinery involved in responses to shear stress.

### Cytoskeleton

The cytoskeleton comprises three types of polymers: microtubules, microfilaments composed of actin and myosin, and intermediate filaments (IFs) composed of vimentin, keratins and desmin. One of the main functions of the cytoskeleton is to maintain cell shape. To do that, the fibers can exert tension that opposes external mechanical cues, creating resilience to environmental mechanics but also allowing the system to immediately respond to any internal or external mechanical stress. Consequently, some of the major changes observed upon response to shear stress arise from modification of the cytoskeleton (Fig. 2). A common feature of a cytoskeleton adapted to shear stress is the presence of stress fibers (contractile actomyosin filaments) that are aligned in the flow direction, contributing to atheroprotection (see Glossary) (Galbraith et al., 1998; Noria et al., 2004); however, other related phenotypes have been found. For instance, endothelial cells exposed to low FSS conditions exhibit fewer central stress fibers than cells under normal physiological conditions (Verma et al., 2015). In contrast, cells under high levels of FSS exhibit thicker and longer central microfilaments, with disruption of the peripheral microfilaments (Colangelo et al., 1998). This change in the organization of microfilaments also contributes to the distribution of FAs, which bind to the most stable actin bundles (see above).

IFs, the most flexible cytoskeletal filaments, are also altered under shear stress. VIFs are the major IFs in endothelial cells and form a fine-meshed network within cells, whereas keratin intermediate filaments (KIFs), found normally in epithelial cells, are more sparsely distributed and are assembled into bundles (Nolting et al., 2014). Shear stress can induce a reorganization of IFs. For instance, endothelial cells exhibit rapid displacement of VIFs during the first minutes of exposure to FSS (Helmke et al., 2000, 2001). In addition, VIFs are able to stabilize cell–cell junctions and apply tension to PECAM-1, suggesting that VIFs are intermediaries in the force transmission between cells (Conway et al., 2013; Sanghvi-Shah and Weber, 2017). On the other hand, FSS can also increase stiffness of the KIF network in the peripheral cytoplasm, a phenomenon mediated by redistribution of KIFs due to stabilization of the filaments by phosphorylation (Flitney et al., 2009; Sivaramakrishnan et al., 2009).

As described above, shear stress induces redistribution of several types of filament, and as these filaments are in a network, any rearrangement of one type of filament induces stretching and compression of neighboring filaments (Huber et al., 2013). Interestingly, stretched semi-flexible filaments (microfilaments and microtubules) exert a greater force than compressed filaments (Broedersz and MacKintosh, 2014). Thus, the response of any filament to a force opposing its mechanical deformation depends on the orientation of the filament relative to the direction of force experienced; this implies that the induction of normal stress (see Glossary) within the cytoskeleton relative to the cell membrane is an expected response to shear stress. In line with this, measurement of the deformation of the different cytoskeletal fibers under FSS has shown that the response of semi-flexible filament networks is indeed induction of normal stress in the direction of the inner side of the polymer (Janmey et al., 2007). In addition, the distribution of semi-flexible filaments under shear stress leads to an inhomogeneous accumulation of energy, which can induce a partial disintegration of the cytoskeleton (Pajic-Lijakovic and Milivojevic, 2022a). Taken together, the sensitivity of a cell to shear stress can be explained by the rheology of its semi-flexible cytoskeletal filaments, such as actin filaments and microtubules, rather than being attributed to the more flexible IFs.

## Shear stress in collective cell migration

During collective cell migration (CCM), cells are submitted to several types of stress resulting from the interaction between the cells and their environment (Barriga and Mayor, 2019; Espina et al., 2022; Friedl and Wolf, 2010). For instance, collective migration means that a group of cells slides over a substrate, generating shear stress in the boundary between the migratory surface and the moving cluster. In addition, some cells within migratory clusters can exchange positions, and these exchanges generate shear stress at the surface these of cells. *In vivo*, cells move between tissues, generating compressive forces on migratory cells. Some of these forces have been shown to be relevant for the normal physiology of migratory clusters. Here, we will focus on some observations made for shear stress.

Analysis of migratory cell monolayers points to a tendency of cells to migrate along the direction of the maximum normal stress, which minimizes the generation of shear stress (Serra-Picamal et al., 2012). Despite this, significant shear stresses can occur, even in such two-dimensional contexts. The magnitude of shear stress in the context of CCM depends on (1) the thickness of the interface, (2) the velocity of migrating cell clusters, (3) the tissue viscosity, (4) the elastic shear modulus of the tissue and (5) slip effects (see Glossary) (AbouAlaiwi et al., 2009; Pajic-Lijakovic and Milivojevic, 2020b). Because measuring shear stress in the context of CCM is technically very difficult, some of the major advances in this area have been obtained based on *in silico* modeling of cell behavior under physiological conditions (see Box 2).

**Box 2. Modeling shear stress in collective cell migration**

For modeling purposes, two categories of migratory cell clusters can be differentiated based on their viscoelastic properties: free or only weakly connected cells undergoing highly coordinated migration, which can be treated as a viscoelastic liquid (Guevorkian et al., 2011; Lee and Wolgemuth, 2011), and strongly connected migrating cell clusters, which correspond to a viscoelastic solid (Doxzen et al., 2013; Pajic-Lijakovic and Milivojevic, 2020a). Shear stress results in short-term stress relaxation (see Glossary) and long-term accumulation of residual cell stress (Marmottant et al., 2009; Pajic-Lijakovic and Milivojevic, 2019, 2020a).

Stress relaxation has been studied by modeling the behavior of different types of viscoelastic materials resembling either free or strongly connected cell clusters. For instance, the Maxwell model, which is suitable for viscoelastic liquids, treats the material as a sequence comprising one purely elastic element connected to one purely viscous element in tandem. This model predicts that when this material is submitted to a constant stress, the strain will increase immediately and proportionally over time. Then, when the object is released, stress decays exponentially over time (Pajic-Lijakovic and Milivojevic, 2021). In contrast, the simplest model to describe a viscoelastic solid is the Zener model (Pajic-Lijakovic and Milivojevic, 2020a). Here, two elastic elements and one viscous element are connected in a complex manner. When this system is submitted to constant stress, the elastic portion of the material will instantaneously deform to some extent, but after that, will continue to deform until approaching a steady-state strain. When the object is then released, the stress decays exponentially, similarly to the Maxwell model (Pajic-Lijakovic and Milivojevic, 2020a).

**Fig. 3.**
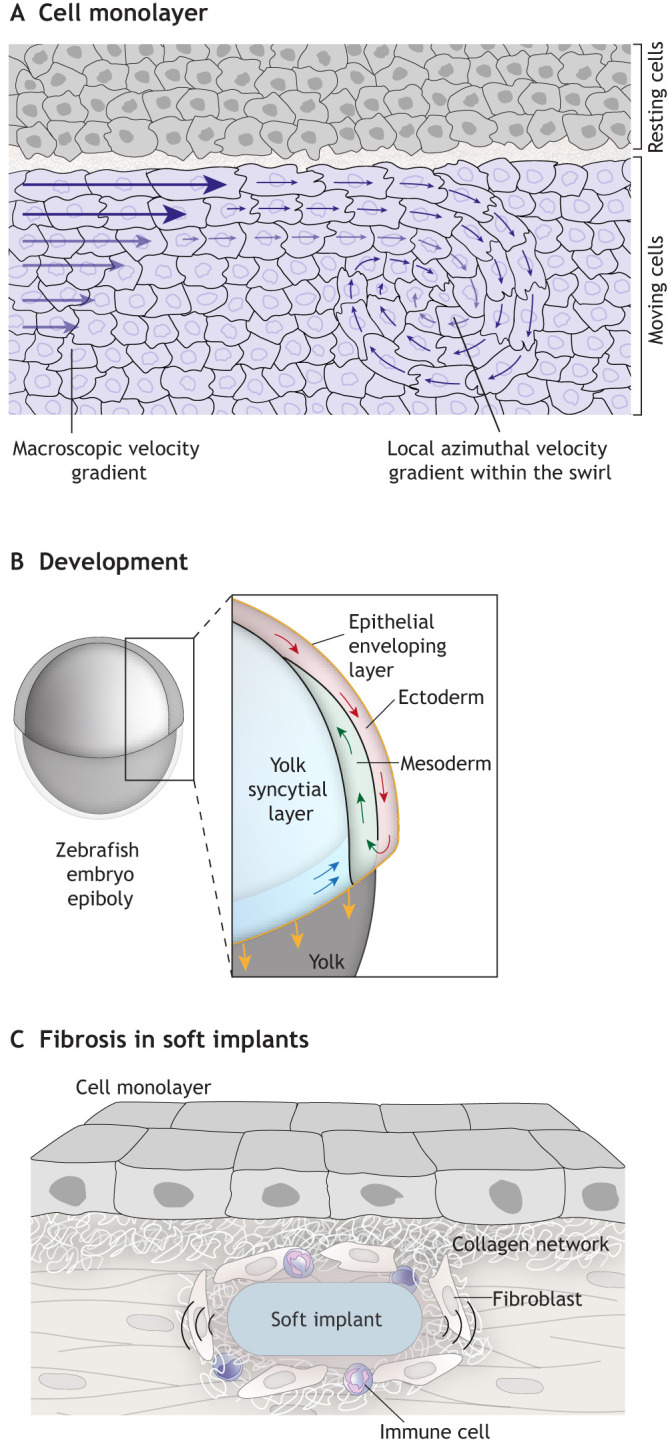
**Shear stress in tissue contexts.** (A) Diagram showing a top view of a cell monolayer sliding over a tissue. Migratory cells have a gradient of velocity from the border to the interior of the cluster. These gradients generate torsion of the velocity vector due to differential friction between migrating cells, in this way starting a swirl. In this context, the local azimuthal shear rate within the cell swirl is lower than the supracellular shear rate generated at the interface, which induces the swirl formation. (B) Schematic illustration of a side view of a zebrafish embryo during gastrulation, with an inset showing associated cell movement. In this case, shear stress is generated at the interface between ectoderm and mesoderm progenitors that migrate in opposite directions. These forces are required to determine the position of the neural progenitors. (C) Diagram showing how fibrosis develops from soft implants. Here, friction leading to shear stress can occur at the boundary between soft implants and surrounding tissues. This shear stress promotes inflammation and deposition of extracellular components in the surrounding connective tissue, leading to fibrosis.

The residual shear stress that accumulates in cells exerts work by generating shear stress torque (see Glossary) against tissue cohesiveness, and so can induce cell swirling motion (CSM) (Pajic-Lijakovic and Milivojevic, 2022b) (Fig. 3A). Since CSM allows single cells within the cluster to reduce the local shear rate, this swirl formation could be a way in which cells minimize shear stress, considering that, as stated above, cells tend to behave in order to minimize shear stress (Serra-Picamal et al., 2012). The appearance of CSM has been studied in several model systems (Lin et al., 2018; Notbohm et al., 2016; Petrolli et al., 2019; Ladoux and Mege, 2017; Vedula et al., 2012). For instance, addressing two-dimensional CCM in confined environments, one study has pointed to reduced tissue cohesiveness accompanied by weak local azimuthal shear rate (see Glossary) and strong contact inhibition of locomotion (see Glossary) as prerequisites for the appearance of the CSM (Lin et al., 2018). Along these lines, weaker cell–cell adhesions and coupling of cellular polarization with local gradients of contractility have been proposed as the mechanisms underlying CSM in confluent monolayers of MDCK cells (Notbohm et al., 2016; Petrolli et al., 2019). In contrast, confluent HaCaT skin cells and Caco-2 intestinal cells form stronger cell–cell adhesion contacts and stiffer monolayers. These cells move collectively in one direction; however, the direction of motion varies over time because the cell clusters form circular trajectories (called ‘incomplete swirls’; Peyret et al., 2019).

Another phenomenon exhibited by migratory cell clusters that depends in the forces within the cluster – either normal or shear – is so-called cell jamming (Sadati et al., 2013). Jamming is a collective process by which granular material from a ‘fluid-like’ state packs and increases its density above a critical value, becoming a ‘solid-like’ material. Cell collectives can be conceived as granular materials, meaning that modification of the physical properties of the collective, such as crowding, adhesion and contractility, can trigger jamming transitions (Pajic-Lijakovic and Milivojevic, 2021; Sadati et al., 2013). Interestingly, it has been shown that applying shear stress to a cluster of relatively free-moving elements that can generate friction between each other can induce different jamming states within the cluster (Bi et al., 2011). However, the contribution of shear stress within a cell cluster to jamming transitions has not been explored. Nevertheless, jamming transitions have been suggested as a means to integrate the possible physical heterogeneities found within a cell cluster in order to explain their behavior in different systems (Chepizhko et al., 2018; Garcia et al., 2015; Sadati et al., 2013).

Developing tissues are constantly remodeling, which requires them to collectively and directionally migrate, either in parallel or against each other, resulting in shear stress (Espina et al., 2023). Some *in vivo* studies have provided insights into how these interactions contribute to shape the early embryo (Friedland et al., 2022; Kale et al., 2018; Smutny et al., 2017). For instance, shear stress generated between axial mesoderm progenitors that migrate in the opposite direction to neuroectodermal cells is essential for the proper positioning of anterior neural progenitors and the overall shape of the embryo (Smutny et al., 2017) (Fig. 3B). This suggests that in order to fully understand processes such as morphogenesis or development, we will need to further study how shear stress arises from tissue interactions and how it acts to shape tissues.

## Shear stress in cancer and fibrosis

Several studies have established a role for FSS in vascular diseases (reviewed in Souilhol et al., 2020; Chistiakov et al., 2017; Baeyens et al., 2016), but here we will focus on the role of shear stress in diseases affecting other tissues. It is well established that the degree of malignancy of cancer cells is determined by the tumor microenvironment, and in this context, shear stress can have an important role in cancer development and behavior (Baghban et al., 2020; Huang et al., 2018; Xun et al., 2023). For example, FSS can increase cancer cell death, regulate tumor proliferation, and promote invasion and metastasis (Huang et al., 2018). Many cancer cell lines exposed to laminar shear stress exhibit increased apoptosis in comparison to cells exposed to oscillatory shear stress or steady media; this effect is mediated by bone morphogenetic protein receptor type-1B (BMPR-1B), BMPR-1B-specific SMAD1 and SMAD5, and p38 mitogen-activated protein kinases in Hep3B cells (Lien et al., 2013). In addition, FSS can lead to reduced proliferation by inducing an arrest at G1-S or G2-M stages of the cell cycle of colon cancer cells and adherent osteosarcoma cell lines (Avvisato et al., 2007; Cheng et al., 2009). Finally, FSS has been shown to promote the activation and expression of a wide range of cytokines and mechanosensitive molecules (Qazi et al., 2013; Wang et al., 2015). For instance, FSS in chondrosarcoma cells can activate VEGFB and VEGFD, leading to cyclic AMP and PI3K activation (Qazi et al., 2013; Wang et al., 2015). This, in turn, promotes the expression of matrix metalloproteinase 12 (MMP12), leading to increased invasive behavior *in vitro* (Wang et al., 2015; Lee et al., 2017). Similarly, prostate cancer cells subjected to FSS induce nuclear translocation of YAP1, a very well-known mechanosensitive transcription factor. In this case, YAP1 activation can induce motility genes, thus increasing cancer metastasis (Lee et al., 2017).

Some medical interventions, such as the presence of implants, can lead to fibrosis due to shear stress. Fibrosis is characterized by the formation of connective tissue attached to the contact surface between the epithelium and other endogenous or exogenous structures. Fibrosis is induced by the immune response of the body and has been proposed to protect the surrounding cells against shear stress (Noskovicova et al., 2021; Henderson et al., 2020), among other forces. Studying shear stress in the context of fibrosis might not only help to further understand how this condition arises in various pathological contexts, but also explain the emergence of fibrosis due to frictional shear stress generated between tissues and soft implants (such as silicone implants, elastomeric clamps and bands, reinforcing polymeric meshes, and contact lenses) (Pitenis et al., 2018) (Fig. 3C). Inflammatory responses to implants arise from shear stress occurring between the soft implant and the surrounding tissue (Noskovicova et al., 2021; Pitenis and Sawyer, 2020). The extent of the inflammatory response, and thus fibrosis, varies between the different implanted materials and can be quantified by the thickness of the fibrous capsule and distribution of collagen. The shear stress observed in these conditions can promote cell movement and influence cell division, as well as provoke immunological responses, which can lead to various diseases. Remarkably, sliding of hydrogel beads on the surface of human corneal epithelial cell monolayers, which generated shear stress of 60 Pa, was sufficient to increase the expression of the pro-inflammatory factors interleukin-1β (IL1B), interleukin-6 (IL6) and matrix metalloproteinase 9 (MMP9), as well as the pro-apoptotic factors DNA damage-inducible transcript 3 (DDIT3) and FAS cell surface death receptor (FAS) (Pitenis and Sawyer, 2020; Pitenis et al., 2018). This is one example, but we are far from a complete understanding of the basis of shear stress-induced fibrosis, both in response to implants and in other scenarios. Thus, further research and the development of tools are needed to fully exploit the biomedical and morphogenetic potential of shear stress.

## Concluding remarks

As discussed above, our current knowledge about the mechanisms and cellular structures involved in sensing, transducing and responding to shear stress mostly comes from cellular systems exposed to a laminar fluid flow. Although these examples have advanced our understanding of shear stress, non-fluid related roles of shear stress, such as friction due to collective migration or tissue morphogenesis, remain comparatively less explored. Thus, further research in these cellular contexts is one of the next challenges in the field. Another question that deserves particular attention is the range at which cells respond to shear stress: why are cells able to respond to such a low threshold of shear stress, compared to, for example, physiological stress? Along the same lines, how do cells dissect shear stress from physiological stress? In this regard, advances in the development of sensors and strategies that allow to dissect these questions would greatly help in fully understanding the role of shear stress in a variety of biological contexts.
